# Multi-scale dissection of wing transparency in the clearwing butterfly *Phanus vitreus*

**DOI:** 10.1098/rsif.2023.0135

**Published:** 2023-05-31

**Authors:** Cédric Finet, Qifeng Ruan, Yi Yang Bei, John You En Chan, Vinodkumar Saranathan, Joel K. W. Yang, Antónia Monteiro

**Affiliations:** ^1^ Biological Sciences, National University of Singapore, 117543 Singapore; ^2^ Engineering Product Development, Singapore University of Technology and Design, 487372 Singapore; ^3^ Ministry of Industry and Information Technology Key Lab of Micro-Nano Optoelectronic Information System & Guangdong Provincial Key Laboratory of Semiconductor Optoelectronic Materials and Intelligent Photonic Systems, Harbin Institute of Technology, Shenzhen 518055, People's Republic of China; ^4^ Division of Science, Yale-NUS College, National University of Singapore, 138609 Singapore; ^5^ NUS Nanoscience and Nanotechnology Initiative (NUSNNI), National University of Singapore, 117581 Singapore; ^6^ Institute of Materials Research and Engineering, A*STAR (Agency for Science, Technology and Research), 138634 Singapore

**Keywords:** transparency, clearwing butterfly, hydrophobicity, structural coloration, Hesperiidae

## Abstract

Optical transparency is rare in terrestrial organisms, and often originates through loss of pigmentation and reduction in scattering. The coloured wings of some butterflies and moths have repeatedly evolved transparency, offering examples of how they function optically and biologically. Because pigments are primarily localized in the scales that cover a colourless wing membrane, transparency has often evolved through the complete loss of scales or radical modification of their shape. Whereas bristle-like scales have been well documented in glasswing butterflies, other scale modifications resulting in transparency remain understudied. The butterfly *Phanus vitreus* achieves transparency while retaining its scales and exhibiting blue/cyan transparent zones. Here, we investigate the mechanism of wing transparency in *P. vitreus* by light microscopy, focused ion beam milling, microspectrophotometry and optical modelling. We show that transparency is achieved via loss of pigments and vertical orientation in normal paddle-like scales. These alterations are combined with an anti-reflective nipple array on portions of the wing membrane being more exposed to light. The blueish coloration of the *P. vitreus* transparent regions is due to the properties of the wing membrane, and local scale nanostructures. We show that scale retention in the transparent patches might be explained by these perpendicular scales having hydrophobic properties.

## 1. Introduction

Animal transparency, i.e. the physical state of letting light propagate through the body, is, arguably, the ultimate mechanism of camouflage [1]. Nearly transparent bodies are widespread in pelagic organisms such as jellyfish, sea angels, squids and fish [2,3]. Animal transparency is rare on land, yet we can cite a few instances in insects: the wings of most insects as well as those of glasswing butterflies [4], the prothorax and elytra margins of tortoise beetles [5] and the pupal case in many butterfly species just before eclosion [6]. In addition, cases of translucency have been described in amphibians, such as the glass frogs, the Barton Springs salamander and some tadpoles [7]. Translucency, which results in only partial transparency as a result of scattering from heterogeneous materials and interfaces [8], acts as modifiable camouflage by decreasing edge diffusion [9].

The wings of butterflies and moths (Lepidoptera) are a good model to study the development, the ecological function(s) and the evolution of transparency from a non-transparent ancestral state. Wing transparency evolved several times independently during the diversification of Lepidoptera from ancestors with opaque wings [4]. Lepidopteran wings are typically covered with chitinous scales that bear colour produced by pigments [10,11], photonic nanostructures [12] or a combination of both [13–15]. Beyond their role in coloration, wing scales also play a role in thermoregulation [16,17] and water repellence [18]. As such, making wings transparent involves changing the development of scales, and potentially also their biological role.

Wing transparency can be achieved by a greater exposure of the wing membrane in different ways. Lepidoptera can simply lose scales [19–22], replace the typical flat, paddle-shaped scales by thin, hair-like piliform scales [18,23,24] or reduce pigmentation in the exposed membrane [4]. The replacement of archetypal, flattened scales with piliform ones has been thoroughly investigated in the glasswing butterfly *Greta oto*, which also uses surface waxes in the exposed membrane to reduce glare [25]. However, little is known about other potential ways of achieving transparency.

In the present study, we explore the structural basis and the function of the wing transparency in the musical ghost-skipper, *Phanus vitreus*, a clearwing butterfly in the family Hesperiidae, primarily found in lowland rainforests from Mexico to southern Brazil [26]. This species is remarkable for having evolved transparent wing patches while retaining scales in these patches [4], and exhibiting a unique visual effect due to the blue/cyan coloration of the transparent zones. The retention of scales suggests that they may have an important functional role. We applied optical and scanning electron microscopy (SEM), as well as focused ion beam (FIB) milling to characterize the structure of both the wing membrane and the wing scales in the transparent area. By combining UV–visible–near-infrared microspectrophotometry and optical modelling, we found that the transparent wing regions reflect blue and cyan light, and we disentangled the relative contribution of the wing membrane, the scales and the scale nanostructures to the overall coloration of the wing. Furthermore, contact angle goniometry showed that the modified scales of the transparent wing regions play a role in water repellence.

## 2. Results

### 2.1. Characterization of the transparent regions of the *Phanus vitreus* wing

**Figure 1.**
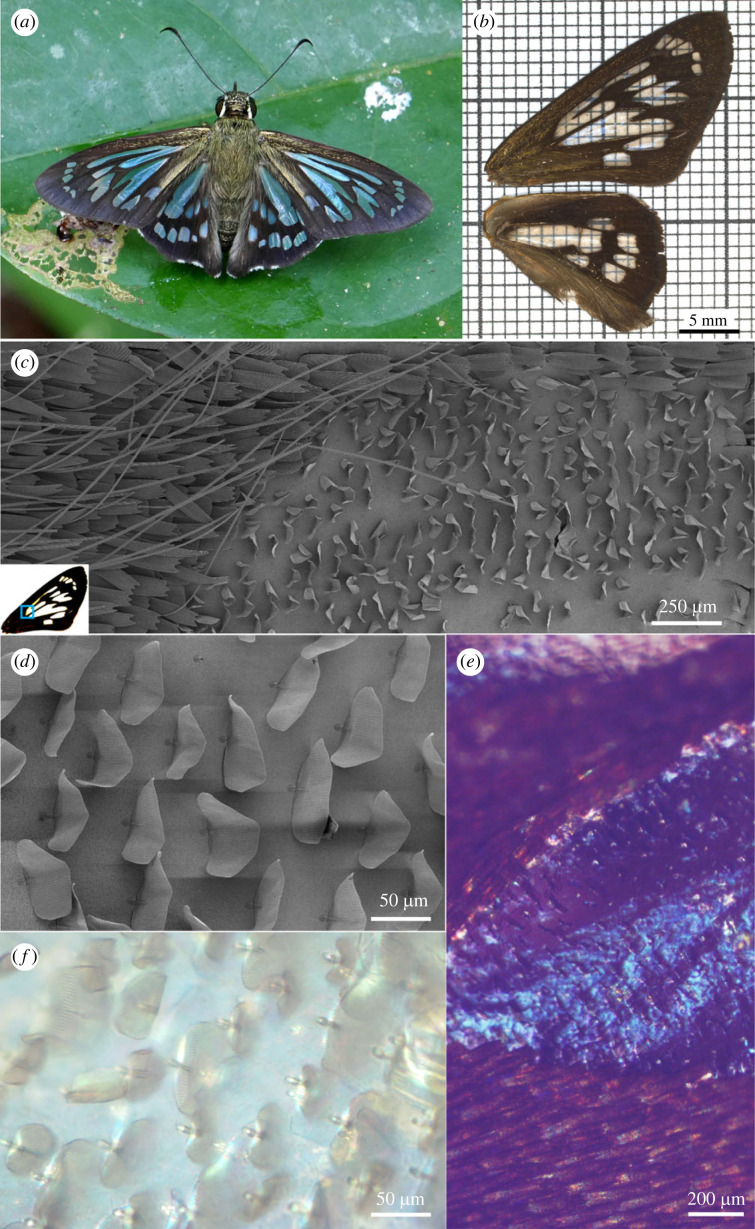
Multi-scale characterization of the clearwing musical ghost-skipper *Phanus vitreus*. (*a*) Adult *P. vitreus* beside Lake Soledad, Madre de Dios, Peru (photo: courtesy of Les Catchick). (*b*) Right fore- and hindwings mounted on graph paper. The grid is visible through the transparent windows of the *P. vitreus* wing. The transparent regions display a light brownish coloration. (*c*) SEM image of the edge between dark (left) and transparent (right) wing regions. The dark region is densely covered with flat, long and pigmented scales, whereas the transparent region shows sparse, small and nearly vertically arranged scales. Inset: the blue square highlights the wing area observed by SEM. (*d*) SEM close-up of the scales from the windows. (*e*) Epi-illumination of a transparent wing region mounted on carbon tape. (*f*) Transparent wing region mounted on a glass slide and examined with transmitted light.

The *P. vitreus* wing pigmentation pattern consists of a black background covered with pigmented scales, and several transparent ‘windows' on both the forewing and the hindwing (figure 1*a*,*b*). The windows are not perfectly transparent, but rather show a light blue-green reflection (figure 1*a*), and a slight brownish tint when placed against a white background (figure 1*b*). The mean light transmittance is 85% in the visible regime under normal incidence (electronic supplementary material, figure S1). The transparent areas contain only one type of sparse scales, very short in length (approx. 40 µm; figure 1*c*), and different from the flanking cover and ground black scales. These short scales have fused upper and lower laminae that are reminiscent of the fused scales of primitive Lepidoptera [27–29]. Contrary to the majority of butterfly scales, the scales of the transparent areas in *P. vitreus* do not lie flat on the wing, but are attached almost perpendicular to the wing surface (average insertion angle of 80 ± 5°) (figure 1*c*,*d*). When mounted on a black background and epi-illuminated, the wing membrane of the windows appears blue (figure 1*e*). When mounted on a glass slide and trans-illuminated, the same wing membrane shows an overall cyan hue (figure 1*f*). These observations prompted us to also investigate the basis of this blue–cyan coloration, in addition to the mechanisms of overall transparency.

### 2.2. Wing membrane of the windows: ultrastructure

**Figure 2.**
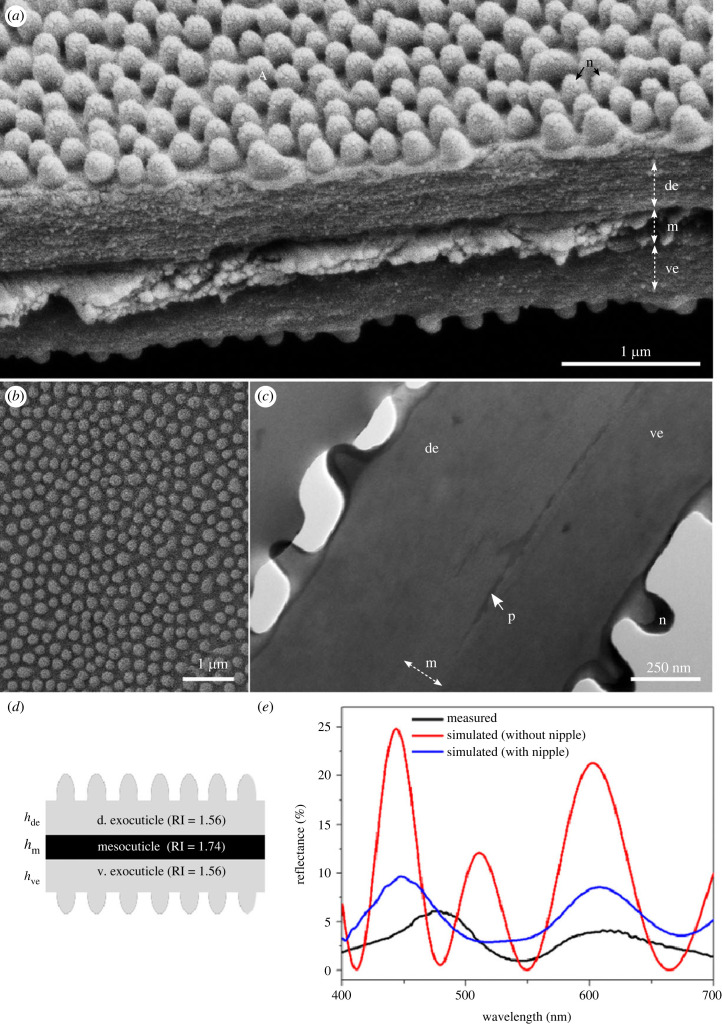
Structure and optical properties of the *Phanus vitreus* window wing membrane. (*a*) SEM close-up of a naturally torn transparent region of the wing. Two main cuticular layers are visible: the exocuticle and the mesocuticle. (*b*) SEM close-up of the wing membrane surface showing the regular arrangement of nipple array nanostructures. (*c*) TEM cross-section of the same sample without staining. (*d*) Best-fit optical model. (*e*) Measured and simulated reflectance. de, dorsal exocuticle; m, mesocuticle; n, nipple nanostructure; p, pigment; ve, ventral exocuticle.

To examine the mechanism of wing transparency, we first characterized the inner structure of the wing membrane by electron microscopy. SEM cross-sections of a torn wing showed that the wing membrane can be divided into three main cuticular layers: a dorsal and ventral exocuticle, and a mesocuticle as the middle layer (figure 2*a*). The exocuticle consists of a nipple array nanostructure (figure 2*b*), without wax, overlaying a lamellar organization (figure 2*c*). The nipple array has an average height of *h*_n_ = 142 ± 10 nm and an average diameter of *d_n_* = 135 ± 11 nm (figure 2*a*). The nipple array is randomly arranged with an average pitch of 300 nm (figure 2*b*; electronic supplementary material, figure S2). SEM images did not show the presence of wax-based nanopillars on top of the nipple nanostructures described in the glasswing butterfly [25]. Nevertheless, we tested for any contribution of epicuticular wax to the wing membrane reflectance. We manually removed the scales by softly brushing a transparent wing region with a paintbrush (electronic supplementary material, figure S3), followed by plasma cleaning to remove surface hydrocarbons (electronic supplementary material, figure S4) [30]. Then, we measured the reflectance of the treated wing membrane. No significant difference was detected between the treated and untreated samples, suggesting that epicuticular wax does not play a role in the optical properties of the wing membrane in *P. vitreus* (electronic supplementary material, figure S5). Under the nipple array, the alternation of bright and dark layers, which is also visible in unstained samples (electronic supplementary material, figure S6A), likely results from a patterned distribution of pigment. The inner mesocuticle is made of a less dense material deposited perpendicular to the exocuticle (figure 2*a*). In the centre of this mesocuticle lies an electron-dense layer (visible in TEM) (figure 2*c*), whose persistence in unstained samples suggests its pigmentary nature (electronic supplementary material, figure S6A).

The pigments present in the wing membrane, and which explain its pale brownish coloration (figure 1*b*), are likely melanins. This was confirmed by immersing wings in warm 3% H_2_O_2_ overnight, which breaks down melanin [31,32]. After H_2_O_2_ treatment, the wing membrane was less pigmented and exhibited a reduced visible-light absorbance (electronic supplementary material, figures S6B and S6C).

### 2.3. Wing membrane of the windows: photonics

In order to investigate how the nipples and the overall membrane structure impacted wing transparency, we used a modelling approach. Our model consists of a two-dimensional nipple array-based chitinous exocuticle with dimensions obtained from our TEM images (figure 2*d*). The nipple nanostructures were modelled as half ellipsoids with an average height of *h*_n_ = 142 ± 10 nm, an average diameter of each nipple determined as *d*_n_ = 135 ± 11 nm, and an average pitch (distance between two nipples) of 300 nm. We modelled the dorsal exocuticle with an average thickness of *h*_de_ = 436 ± 20 nm, the pigmented mesocuticle with an average thickness of *h*_m_ = 194 ± 7 nm and the ventral exocuticle with an average thickness of *h*_ve_ = 412 ± 10 nm, as per our direct measurements. Assuming that cuticle is mainly made of chitin, our optical model uses the refractive index of chitin *n* = 1.56 for both the dorsal and ventral exocuticles [33,34]. Estimating the refractive index of the pigmented mesocuticle required to consider the effect of absorbing pigments. Light propagation in absorbing materials can be described using a complex-valued refractive index. The imaginary part explains the attenuation, while the real part accounts for refraction. We estimated the wavelength-dependent imaginary part of the refractive index from our measured absorption spectrum as previously described [35]. The imaginary part is quite small (less than 0.012 in the visible range; electronic supplementary material, figure S7), which makes the transparent wing possible. The real part of the refractive index of the pigment layer is 1.74, similar to previously reported values [36].

We then ran alternative models that differed in the inner structure of the wing membrane to examine which aspects of the membrane morphology altered light reflectance (hence transparency), and to pick the best-fitting model. The ‘one-layer' model assumed a homogeneous cuticular content of the wing membrane, and led to a reflectance spectrum with multiple peaks that differed from the bimodal spectrum obtained experimentally (electronic supplementary material, figure S8A). The ‘three-layer' model assumed the presence of melanin in the mesocuticle and considered the wing membrane as being slightly asymmetrical, with dorsal exocuticle thicker than ventral exocuticle. The simulated reflectance data using this model provided a better fit to the experimental reflectance data (figure 2*e*). A model where the ventral and dorsal exocuticle had the same thickness produced very similar results (electronic supplementary material, figure S8B), showing that the slight asymmetry of the membrane does not play a crucial role in its reflection. Comparing the previous ‘three-layer' model *in silico* with a model in which the nipple nanostructures were absent, we obtained very similar spectral reflectance signatures, such as the position and shape of the peaks, but the reflectance intensity was significantly lower in the nipple model (figure 2*e*). This demonstrates that the nipple nanostructures decrease the reflectivity of the wing membrane, making the wing more transparent. The discrepancy between the simulated reflectance spectrum of the model with nipples and the measured reflectance spectrum could be ascribed to the non-uniformity of the cuticular thickness and nipple shape in the real wing membrane. Moreover, we plotted the simulated transmittance spectrum of the model with nipples (electronic supplementary material, figure S9). The transmittance value in the visible range is larger than 80%, which is consistent with the transparent appearance of the wing. We also simulated the electric field distributions for the ‘three-layer' model with nipples (electronic supplementary material, figure S10). The electric field magnitudes above the light source at the peak/dip wavelengths of 440 nm, 543 nm and 607 nm are 0.34, 0.22 and 0.36, respectively.

### 2.4. Modified scales of the windows produce structural colour

We next looked at how the retention of scales on the wing membrane impacted wing membrane reflectance. We measured the spectral reflectance of both the intact transparent wing area and the same region after the removal of the scales. Reflectances were similar, but the intensity of the second peak (approx. 600 nm) was slightly reduced when the scales were removed (electronic supplementary material, figure S11). Thus, scales contribute to the overall reflectivity of the wing in *P. vitreus*, but their contribution at normal incidence is limited given their nearly upright orientation.

**Figure 3.**
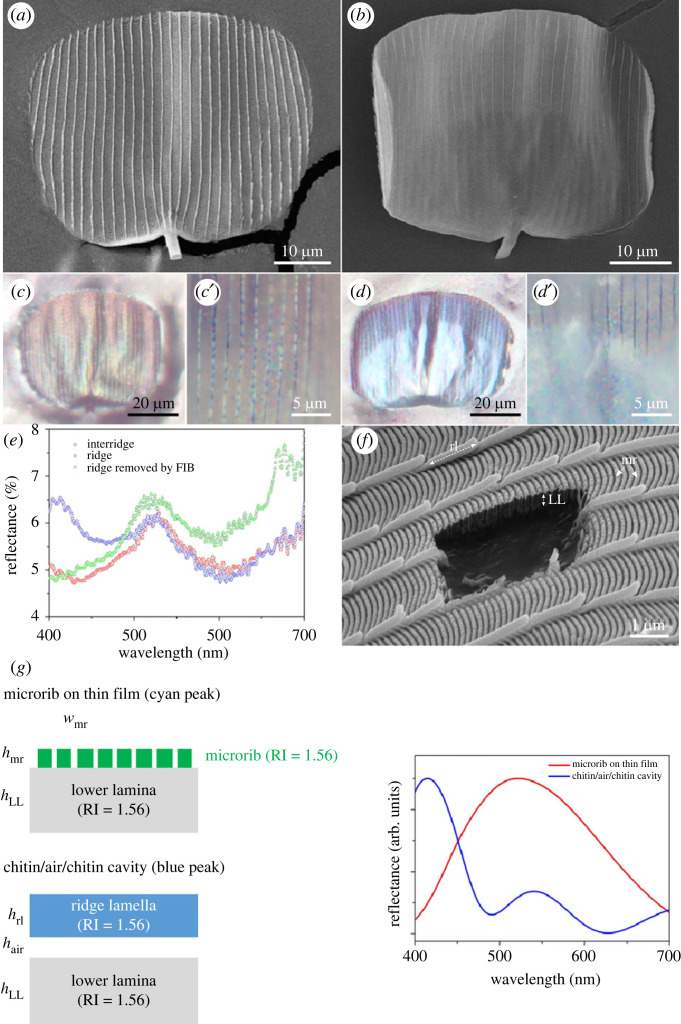
Structure and optical properties of the *Phanus vitreus* window scales. (*a*) SEM image of the abwing (upperside) surface and (*b*) SEM of the adwing (underside) surface of a scale. (*c*) Optical microscopy image of the abwing surface under epi-illumination using a 20× objective. (*c*′) The same scale under a 100× objective. (*d*) Optical microscopy image of the adwing surface under epi-illumination using a 20× objective. (*d*′) Same scale under a 100× objective. (*e*) Reflectance measured on different areas of the scale: the ridge (in blue), the ridge after its milling (in green), and the domain covered with microribs between two consecutive ridges, or the interridge (in red). (*f*) FIB–SEM cross-section of the scale. (*g*) Best-fitting optical models for the ridge and inter-ridge colorations, and resulting simulated reflectance. LL, lower lamina; mr, microrib; rl, ridge lamella.

To investigate the contribution of the different hierarchical nanostructures of the scale to overall wing reflectivity, we examined the scales with SEM, FIB–SEM and optical imaging. The abwing (upperside) surface of the scale consists of a grid of longitudinal chitinous ridges (figure 3*a*), whereas the adwing (underside) surface is largely featureless (figure 3*b*). Under epi-illumination, the abwing surface displays a predominant cyan hue with local yellow and orange tints (figure 3*c*). At higher magnification, the ridges show blue dots regularly spaced along their length (figure 3*c*′). The adwing surface appears bluer, with a Pointillist multi-coloured effect being visible at higher magnification (figure 3*d*′; electronic supplementary material, figure S12). Taken together, our observations suggest that the predominant cyan hue of the scale comes from the interridge region of the scale, and the deeper blue coloration comes from the ridge.

To test these hypotheses further, we measured the reflectance of each region of the scale with a microspectrophotometer. Whereas the reflectance spectrum of the interridge region shows a single peak around 520 nm, the reflectance spectrum of the ridge region comprises two peaks: centred around 410 and 520 nm (figure 3*e*). Because the minimum sample area of the microspectrophotometer (1 µm × 1 µm) exceeds the width of the ridge (approx. 250 nm) and given the high numerical aperture of the objective (0.9), it is likely that the ridge measurement collected the light reflected from both the ridge and the surrounding interridge regions.

To further ascertain the effect of the ridge and interridge regions on scale colour production, we selectively removed a part of the ridge by FIB–SEM on an uncoated scale, then remeasured the reflectance of the milled ridge area. The first peak (approx. 410 nm) disappeared, whereas the second peak (approx. 520 nm) remained (figure 3*e*), suggesting that the ridge and the interridge region, respectively, give rise to the first and second peaks.

To further validate the structural basis of the scale coloration, we ran optical simulations. First, we built a simple optical model of the interridge region that consists of microribs (average thickness *h*_mr_ = 45 ± 2 nm, average width *w*_mr_ = 62 ± 4 nm, average distance between two microribs *d* = 58 ± 3 nm) on top of a lower lamina (average thickness *h*_LL_ = 225 ± 12 nm) (figure 3*g*). The resulting simulated reflectance shows a single peak around 520 nm, whose width is bigger (i.e. colour less saturated) than the one obtained for experimental measurements. We also investigated the impact of the distance between microribs on the reflectance by varying the microrib spacing in our model. We found that the reflection spectrum was blue-shifted towards shorter wavelengths when the distance between microribs was increased (electronic supplementary material, figure S13).

We then built an optical model of the ridge itself. The ridge consists of partly overlapping lamellae whose tip stands over an air layer (figure 3*f*). Under epi-illumination, the lamella tips exhibit a blue coloration, leading to the dotted appearance of the ridge (figure 3*c*′). We found that a multi-film interference model made of alternating materials with different refractive indices (*n*_air_ = 1, *n*_chitin_ = 1.56), recapitulates the empirical reflectance data. In our model, the top chitinous layer (the lamella) is separated from the bottom chitinous layer (the lower lamina of the scale) by a distance that corresponds to the average thickness of the air layer, *h*_air_. The simulated reflectance shows a peak around 410 nm (figure 3*g*) that recapitulates the reflection measured for the ridge area (figure 3*e*).

### 2.5. Modified scales contribute to the hydrophobicity of the windows

**Figure 4.**
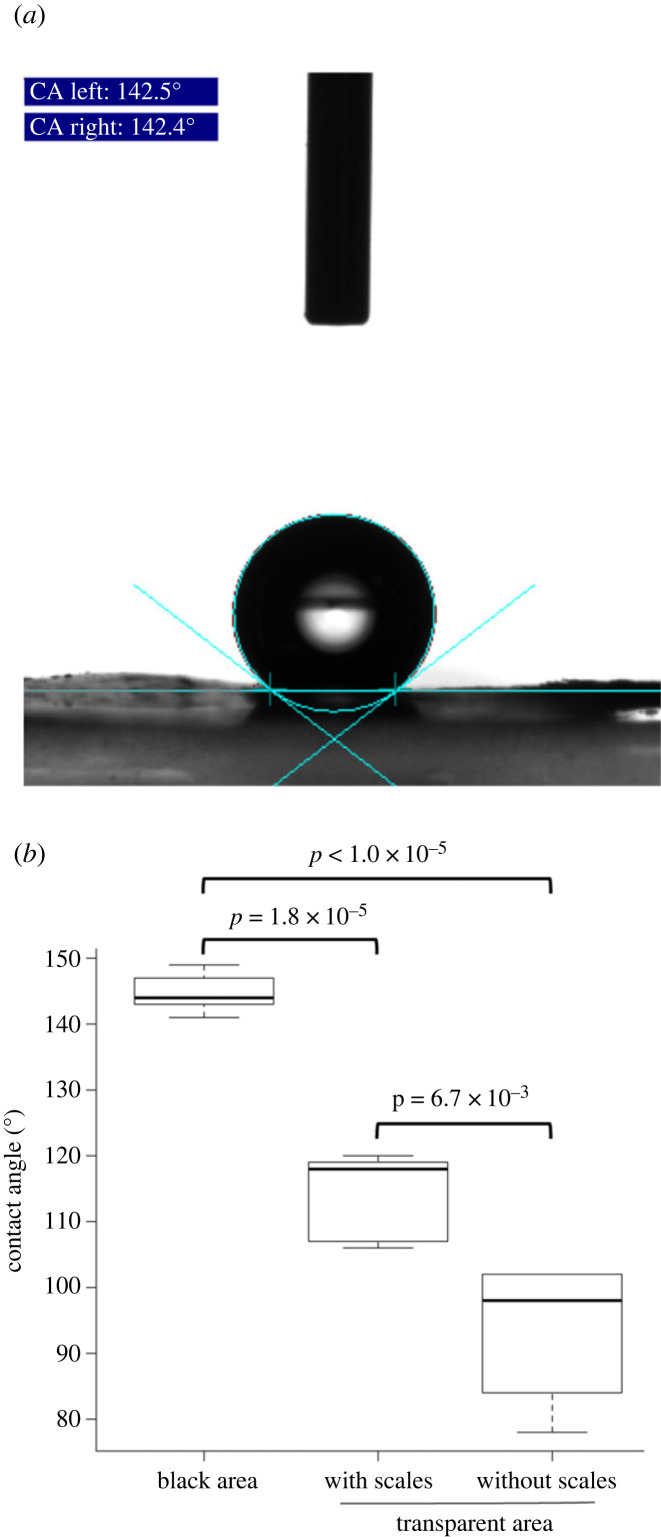
Contact angles on different areas of the *Phanus vitreus* wing. (*a*) Experimental set-up: a droplet of water is deposited on the wing by a capillary (visible on the image), then the baseline is manually defined and the geometry and angles of the droplet are extracted by the software. The present case shows an example where the left contact angle is 142.5° and the right angle is 142.4°. (*b*) Maximal contact angle is obtained for dark regions of the wing (*θ*_mean_ = 144.8°) that are therefore nearly superhydrophobic. Transparent regions of the wing are less hydrophobic than dark regions (*θ*_mean_ = 114°), and become significantly less hydrophobic when devoid of scales (*θ*_mean_ = 92.8°).

The inclusion of these vertical-oriented scales increases the overall reflectivity of the wing membrane, making it less transparent, so it is unclear why scales are retained at all in the transparent patches. In order to understand their putative biological function, we investigated the contribution of the vertical scales to the overall hydrophobicity of the transparent wing area. To do so, we placed a droplet of water on the wing surface and measured the contact angle of the droplet with the wing surface (figure 4*a*). The capability of a substrate to repel water (i.e. hydrophobicity) increases with increasing contact angles. A substrate is termed hydrophobic when the contact angle exceeds 90°, and superhydrophobic for values greater than 150° [37]. We first assessed the hydrophobicity of the wing area covered with normal black scales and found an average static contact angle (°) of 145 ± 3.2, which is highly hydrophobic (figure 4*b*) and comparable to values reported in other species [24]. Then we did the same calculations for the transparent region and found a significantly smaller average static contact angle of 114 ± 6.9 (*t*-test, d.f. = 4, *p*-value = 1.8 × 10^−5^). This value, however, is significantly more hydrophobic than the value of the membrane after manually removing the scales: 93 ± 11.1 (*t*-test, d.f. = 4, *p*-value < 6.7 × 10^−3^) (figure 4*b*). We conclude that retaining scales in the transparent area improves overall wing hydrophobicity.

## 3. Discussion

### 3.1. Evolving sub-optimal wing transparency

Wing transparency has evolved multiple times independently across Lepidoptera [4], suggesting the ecological relevance of this trait. In Lepidoptera, transparency might play a role in thermoregulation as evidenced by the decreasing transmittance of the transparent wing area as latitude increases in a large sample of species [4]. Transparency also serves as camouflage by allowing the resting background colours to emerge through the wings of diurnal butterflies [4,38,39] and nocturnal resting moths [40]. Furthermore, transparency has been shown to act as an aposematic signal among co-mimetic species [41].

Although the ecological relevance of wing transparency remains unknown in *P. vitreus* butterflies, the multiple mechanisms deployed to optimize this trait suggest important selective pressures on the evolution of transparency in this species and perhaps other lepidopterans. The nearly vertical orientation of the fairly short scales maximizes the exposure of the wing membrane to light. However, the wing membrane can still reflect light due to the stark difference in refractive index between air (*n* = 1) and chitin (*n* = 1.56) [42]. Light reflectance is reduced, however, via the nipple array on the surface of the wing membrane, which increases light transmission through the tissue. This strategy, as well as the deployment of wax nanopillars and conical protuberances to reduce light reflectance, have previously been observed in the wings of other species of butterflies [25,41,43–45], in the wings of moths [20,46], in the wings of cicadas [47,48] and in the eyes of moths [49,50] and other insects [51–53]. These natural strategies have also inspired the development of anti-reflective coatings [54–57].

Our findings, however, show that the windows on the *P. vitreus* wing deviate from perfect transparency. The light is not entirely transmitted through the wing, but partly absorbed by the pigment in the wing membrane and partly reflected by the encountered scale nanostructures. Interestingly, published studies of the glasswing butterfly *G. oto* do not mention the presence of pigment in the wing membrane, nor optical models that necessitate a pigmented wing membrane to fit the measured reflectance [25,43]. Future studies might help to determine whether the pigmentation of the *P. vitreus* wing membrane is a secondarily acquired trait, or alternatively, an ancestral feature in Lepidoptera that has been lost in other transparent species like *G. oto*. Another difference between these two species is that *P. vitreus*, in contrast to *G. oto*, shows no wax-based nanopillars on top of the nipple nanostructures. It has been shown that these wax-based nanopillars, mainly composed of long-chain *n*-alkanes, reinforce the anti-reflective properties of the nipples in *G. oto* [25] and in other butterflies [41]. Despite this difference in nanopillar composition, the transparent wings have a similar transmittance under normal incidence in *P. vitreus* (85%) and in *G. oto* (84%) [43].

### 3.2. Retaining scales in the transparent wing windows

The strategy ‘zero scales' has evolved several times independently during the evolution of clearwing moths and butterflies [4]. This strategy, which seems rather ultimate and optimal, is however not the rule among clearwing lepidopterans with the majority of species retaining scales in the transparent wing regions [4]. A proximal explanation for such diversity is that different molecular mechanisms are responsible for the modification of the scales among clearwing lepidopterans. Whereas altered neural precursor formation would presumably lead to the absence of scales [58], as is the case for absence and variation in the location of homologous sensory bristles across insects [59–63], the perturbation of more downstream developmental genes would affect the morphology and orientation of the scales [15,64]. An ultimate, adaptive hypothesis would be that retaining scales in transparent wing regions aids in generating structural colours [12], self-cleaning [65], aerodynamics [66] and thermoregulation [67]. Here, we demonstrate that the upright scales of the transparent regions in *P. vitreus* confer increased hydrophobicity to the wing, a function that might be generalized to most clearwing butterflies and moths [68].

### 3.3. Wing membrane and scales are coloured via thin-film interference

The transparent windows of *P. vitreus* have a lightly iridescent, blue–cyan coloration because the blue–cyan light is mainly reflected by the encountered nanostructures. We have shown that multi-layered thin-film interference explains the coloration both of the wing membrane and of the scale. Our findings show that the wing scales are able to produce structural colours when they stand horizontally under normal light incidence. Even if the nearly vertical and natural position of the scales partly annihilates this coloration, the scales still contribute to the overall coloration of the transparent windows in *P. vitreus*. Furthermore, it is noteworthy that *P. vitreus* belongs to the subfamily Pyrginae, whose representatives are known for basking with their wings held wide open, compared to the half-open wing basking position favoured by other hesperids, suggesting the putative ecological relevance of wing coloration in this species.

Alternatively, these blue hues might be used not for crypsis but for other biological functions. In the pipevine swallowtail butterfly, iridescent blue wing coloration is recognized as a warning signal by avian predators [69]. In *Morpho* butterflies, blue species have usually a fast and/or erratic flight that makes them difficult to locate and catch for birds. Blue in *Morpho* may serve as an escape aposematism by informing predators about the cost of an attack rather than toxicity [70,71]. Blue colours could also be used in mate recognition and choice in *Heliconius* butterflies [72]. Future ecological studies are required to understand the function of the blue–cyan colour in *P. vitreus*.

## 4. Material and methods

### 4.1. Biological samples

Dried specimens of *Phanus vitreus* were obtained from Lepidoptera Exchange (https://lepidopexchange.com/). The individuals were collected in San Vicente, Argentina in November 2007.

### 4.2. Optical imaging

Epi-illumination microscope images were obtained with either a 20× or 100× objective of a uSight-2000-Ni microspectrophotometer (Technospex Pte. Ltd, Singapore) and a Touptek U3CMOS-05 camera.

### 4.3. Scanning electron microscopy

Samples were mounted on carbon tape, and sputter-coated (JEOL JFC-1600) with platinum for 100 s at 40 mA. Samples were imaged using a FEI Versa 3D with the following parameters: voltage 10 kV, current 23 pA. Cross sections of wing scales were obtained by FIB milling using the gallium ion beam of the FEI Versa 3D with the following parameters: beam voltage 8 kV, beam current 12 pA, tilt 52°. Milled samples were imaged as previously described. Thickness and other geometries were measured using the Line tool implemented in Fiji [73].

### 4.4. Transmission electron microscopy

Adult wings were fixed in 2.5% glutaraldehyde in phosphate-buffered saline (PBS, pH = 7.4) for 4 h at 4°C. Wings were washed in PBS and post-fixed in 1% osmium tetroxide for 30 min. Samples were then dehydrated in ethanol and embedded in epoxy resin. Samples were sectioned on a Leica UCT ultramicrotome, stained with lead citrate and imaged with a JEOL JEM-1220 TEM at 100 kV. Thickness and other geometries were measured using the Line tool implemented in Fiji [73]. Ten independent measurements were taken and averaged.

### 4.5. Microspectrophotometry

Samples were mounted on carbon tape. Reflectance spectra with a usable range of 350–950 nm were acquired with a microspectrophotometry set-up using a mercury–xenon light source (Thorlabs, New Jersey, USA) connected to a uSight-2000-Ni microspectrophotometer (Technospex Pte. Ltd, Singapore), using a polished aluminium mirror as a light reference. The microscope's Nikon TU Plan Fluor objectives have the following specifications: 4× (NA = 0.13), 20× (NA = 0.5), 100× (NA = 0.9). Each measurement was averaged 10 times over an integration time of 100 ms. Reflectance spectra from three measurements taken at different locations on the same sample were averaged to account for any variability.

Transmittance spectra were measured using the same set-up, except that the samples were mounted on a glass slide. For reference, we used the light transmitted through the glass side. To obtain absorption spectra, the samples were mounted on a glass slide and covered with a coverslip, and immersed in clove oil as a refractive index matching medium for chitin. A transparent area of the glass substrate covered with clove oil was used as the reference. Transmittance and absorption spectra were obtained by averaging three measurements taken at different locations on the same sample.

### 4.6. Optical simulation

The electromagnetic simulations were conducted with a finite-difference time-domain software (Lumerical Solution Inc.). Chitin was modelled as a lossless material with a refractive index of 1.56. For the reflection spectra, a broadband plane wave was normally incident to the simulated membranes and scales. We used a periodic boundary condition in the *x*/*y*-direction and a perfectly matched layer-absorbing boundary condition in the *z*-direction to absorb light outside the structure regions. Energy monitors were placed behind the light source to record the calculated power flux and obtain the simulated reflection spectra. The electric field profiles in the plane of incidence at the peak/dip wavelengths were recorded by a field profile monitor.

### 4.7. Plasma cleaning

To remove surface hydrocarbons, we cut the wing into small pieces, manually removed the scales from the transparent region, and placed them in a plasma cleaner. Ultraviolet light generated in the plasma breaks most organic bonds of hydrocarbons [30]. Moreover, the radical oxygen species generated react with the broken-down organic contaminants to form mainly water and carbon dioxide that are continuously removed from the chamber. The samples were oxygen-plasma treated using a power of 60 W for durations of 20 and 120 s and a frequency of 13.56 MHz (PICO System, Diener Electronic, Ebhausen, Germany). The oxygen flow rate was set at 15 standard cubic centimetres per minute (sccm). Reflectance was measured subsequently on three independent samples.

### 4.8. Contact angle measurement

In order to measure wing hydrophobicity, we calculated the static contact angle of small droplets of water placed on the surface of the wing. We performed five measurements on non-overlapping transparent regions of the dorsal forewings, on the same transparent region after we manually removed the scales, and on black wing regions. Measures were acquired with an OCA40 Micro system (DataPhysics Instruments GmbH, Filderstadt, Germany) at 25°C with a defined drop volume of 1 µl of ultra-pure water. A video was recorded after the drop was dispensed, and then we used SCA20 software (DataPhysics Instruments GmbH, Filderstadt, Germany) to calculate the static contact angle. Statistical analysis and boxplots were performed using R statistics package version 3.5.0 (the R Project for Statistical Computing, https://www.r-project.org).

## Data Availability

The raw data are available on Zenodo (https://doi.org/10.5281/zenodo.7824043) [74]. The data are provided in electronic supplementary material [75].
